# Highly sensitive protein detection by biospecific AFM‐based fishing with pulsed electrical stimulation

**DOI:** 10.1002/2211-5463.12253

**Published:** 2017-07-10

**Authors:** Tatyana O. Pleshakova, Kristina A. Malsagova, Anna L. Kaysheva, Arthur T. Kopylov, Vadim Yu. Tatur, Vadim S. Ziborov, Sergey L. Kanashenko, Rafael A. Galiullin, Yuri D. Ivanov

**Affiliations:** ^1^ Institute of Biomedical Chemistry Moscow Russia; ^2^ Foundation of Perspective Technologies and Novations Moscow Russia; ^3^ Joint Institute for High Temperatures RAS Moscow Russia

**Keywords:** atomic force microscopy, detection of low‐abundant proteins, mass spectrometry, protein fishing

## Abstract

We report here the highly sensitive detection of protein in solution at concentrations from 10^–15^ to 10^–18^ m using the combination of atomic force microscopy (AFM) and mass spectrometry. Biospecific detection of biotinylated bovine serum albumin was carried out by fishing out the protein onto the surface of AFM chips with immobilized avidin, which determined the specificity of the analysis. Electrical stimulation was applied to enhance the fishing efficiency. A high sensitivity of detection was achieved by application of nanosecond electric pulses to highly oriented pyrolytic graphite placed under the AFM chip. A peristaltic pump‐based flow system, which is widely used in routine bioanalytical assays, was employed throughout the analysis. These results hold promise for the development of highly sensitive protein detection methods using nanosensor devices.

**Abbreviations**
AFM: atomic force microscopy
BSA: bovine serum albumin
HOPG: highly oriented pyrolytic graphite
MS: mass spectrometry
PTFE: polytetrafluoroethylene
SRM: selected reaction monitoring

Novel methods for protein detection with femtomolar sensitivity are necessary to solve proteomic and medico‐biological problems 1, 2. It has been shown that devices with nano‐sized sensor elements can be successfully used for highly sensitive protein detection. One example of the unprecedented sensitivity is biospecific protein detection in a 5 × 10^−19^ m solution with a probe‐based system 3; another is non‐specific protein detection in a 10^−18^ m solution by mass spectrometric selected reaction monitoring (SRM) 4. Earlier we showed that an atomic force microscope, whose nano‐sized probe dimension is comparable to biomolecular sizes 1, 2, 5, provides high sensitivity of the registration system 6 and can be used effectively for protein detection. The capabilities and advantages of nanosensor devices are, however, restricted by the difficulty of protein delivery to the sensor surface. To obviate this difficulty, special techniques are typically used based on the rapid delivery of protein to the surface of a highly sensitive detection element by means of hydrodynamic forces upon turbulent stirring 4, 7, 8. We have also used turbulent stirring for detection of protein disease markers by means of a combination of atomic force microscopy (AFM)‐based fishing and mass spectrometry (MS) analysis (the AFM–MS method) 8. The main advantages of this method are obvious: it allows the concentration of protein from a large volume onto the AFM chip surface, the registration of single biomolecules (by AFM), and MS identification of fished‐out molecules.

Turbulent flow is not always realizable in some analytical systems (e.g. in microfluidic devices) 9. Therefore, currently used methods employ electric forces to improve the fishing efficiency (e.g. 10, 11). It is most convenient to use an external direct current (DC) electric field in electrochemical devices, since a DC field does not require special procedures and high speed stirring of the protein solution. Usually in this case electric fields with very high strength (several kV·m^−1^) are applied 9. Application of organized high strength fields requires the use of microcapillaries, but the implementation of microcapillary systems is hampered by the difficulty of washing biological fluid components from them.

Earlier in our studies 5, 12 we have shown that the concentration of proteins on the surface may be attained without turbulent stirring or high‐strength fields. We have shown 5 that the concentration is more efficient upon the fast (injection) input of protein solution into the measuring cell. It was found that injection of solution leads to charge generation in the solution (10^−9^ °C), and that an electric potential is induced within the measuring cell. It was demonstrated that without an external electric field, with rapid (as opposed to slow) injection input of diluted protein solution, the fishing is efficient. The high sensitivity of this method was demonstrated by detection of proteins with negative net charge – human serum albumin and human cytochrome *b*_5_ – in 10^−17^–10^−18^ m aqueous solutions. It was shown that an external negative voltage applied to highly oriented pyrolytic graphite (HOPG) hinders the protein fishing. The efficiency of fishing with an external positive voltage was similar to that obtained without applying any voltage. It was also found that the efficiency of protein fishing increased with decreasing protein concentration. The input of solution by injection is, however, difficult to perform in routine bioanalytical practice. In most cases, for solution feed in bioanalytical systems, peristaltic pump‐based flow systems are commonly adopted. The results obtained by us 5 have indicated that in the absence of injection, i.e. upon slow input of the analyzed solution with a peristaltic pump, the protein is not fished out from the solution even at 10^−14^ m concentration. Analysis of solutions with lower concentrations with the use of a peristaltic pump was not performed in that study 5.

The results of our further studies 12 have indicated that the flow system can be used for highly sensitive detection of proteins by the AFM–MS method. In this case, to increase the protein fishing efficiency a pulsed AC electric field can be employed. Pulses of AC voltage had 1 ns rise time, 1 kHz frequency and 500 mV amplitude (nanosecond pulsed AC electric field). It was shown that the efficiency of protein fishing increases upon stimulation with exactly nanosecond pulsed AC electric field 12. In contrast to the pulsed voltage, the constant electric field (without the applied AC electric field) and AC sine wave electric field did not increase the efficiency of the AFM‐based fishing of cytochrome *b*_5_. Employing the flow system, cytochrome *b*_5_ protein was detected in 10^−15^ m solution upon electrical stimulation; the presence of the protein was not registered without the stimulation. Thus, we have shown that weak electric fields can be used for concentration of the protein 12. The use of the electric field does not require employment of microcapillaries – which eliminates the problem of clogging of the microcapillaries in the analysis of biological fluids.

In this study, results are presented for biospecific fishing by a combined AFM–MS method with use of the flow system for analyzed solution input. That is, the analysis scheme widely used in bioanalytical systems and required in medical diagnostics is employed. The data obtained have shown that protein can be biospecifically detected in solution with high sensitivity: the efficient AFM‐based fishing out of the protein onto the modified mica surface and MS identification of this protein are observed at concentrations from 10^−15^ to 10^−18^ m.

In the present research, an approach designed by us earlier for detection of disease biomarkers (such as the core antigen of hepatitis C virus and gp120) and described in detail previously 13, 14 has been applied. Briefly, for protein detection, an AFM chip fabricated from mica with immobilized probe molecules was used. The target protein is fished out from solution onto the surface of this chip (protein fishing). After that, the procedure of washing non‐specifically bound biomolecules from the chip is performed. The probe–target complexes are registered and counted by AFM. Then the proteins that are part of the complexes formed on the surface of the AFM chip are identified by MS.

In the study presented here, molecules of avidin were used as a probe, and biotinylated bovine serum albumin (BSA) served as the target protein. The avidin–biotin dissociation constant is 10^−15^ m ^−1^ 15, so the probe–target interaction was sufficiently strong. Thus, the influence of dissociation of the complexes on the results of the analysis is decreased; in such a model system, the possibility of efficient biospecific fishing with a peristaltic pump‐based flow system was demonstrated.

## Materials and methods

### Chemicals and protein

Biotin‐labeled BSA and avidin were from Sigma‐Aldrich (St Louis, MO, USA). To prepare the initial 10^−6^ m protein solution, accurately weighed lyophilized protein was dissolved in Dulbecco's modified phosphate buffered saline.

Analyzed buffered protein solutions were prepared by serial dilution of the initial (10^−7^ m) protein solution to 10^−15^–10^−18^ m concentration in 10 mm potassium phosphate buffer (pH 7.4) upon intensive agitation in a shaker for 30 min.

1‐Ethyl‐3‐(3‐dimethylaminopropyl) carbodiimide hydrochloride and *N*‐hydroxysuccinimide were from Sigma‐Aldrich. Deionized water purified by the Milli‐Q system (Millipore, Billerica, MA, USA) was used throughout the experiments. Acetonitrile was from Merck (Darmstadt, Germany); peptide calibration standards, trifluoroacetic acid and ammonium bicarbonate were from Sigma‐Aldrich; α‐cyano‐4‐hydroxycinnamic acid and dixydroxybenzoic acid were from Bruker Daltonics (Bremen, Germany); porcine trypsin was from Promega (Mannheim, Germany); and HOPG (ZYH grade) was from NT‐MDT, Zelenograd, Russia. Polytetrafluoroethylene (PTFE) film (ρ = 120 g·cm^−3^, 0.1 mm × 12 mm × 20 m) was from Tevton, Moscow, Russia.

### AFM chips for biospecific fishing

Avidin was covalently immobilized onto amino silane surface using *N*‐hydroxysuccinimide–1‐ethyl‐3‐(3‐dimethylaminopropyl) carbodiimide hydrochloride chemistry according to a procedure described previously 1, 14. For this purpose the solution (10 μL) containing avidin (10^−6^ m) in Dulbecco's modified phosphate buffered saline was dispensed onto a silanized mica surface and incubated for 3 min at 15 °C and 80% humidity. After that, the mica chip was washed in deionized water in a shaker for 15 min at 30 °C and dried. Then, the mica chip was covered with a Teflon film with a hole. The immobilization was controlled by AFM.

### AFM‐based fishing procedure and estimation of fishing efficiency

#### Fishing procedure

The scheme of the AFM fishing set‐up has been described in detail in 5, 16. The modification of the fishing scheme used in this study consisted of the following. The mica chip with immobilized avidin molecules was placed onto freshly cleaved HOPG. Then the AFM chip was coated with PTFE film with a small hole (~ 100 μm in diameter, area ~ 8000 μm^2^). Thus, the main part of the surface was isolated, and in the hole of the film a small sensor area was formed. Then, the plate was pressed with a fluoroplastic cuvette (500 μL) so that the sensor area was the bottom of the measuring cell. The diameter of the bottom hole of the cuvette was 2 mm, and the sensor area was centered in regard to this hole.

Atomic force microscopy‐based fishing was carried out using 100 mL of aqueous protein solution. A standard peristaltic pump was employed to feed the solution into the cell. Sterile silicon pipes with the inner diameter ~ 2 mm were used to deliver the solution into the cell. In this case the solution feed rate made up ~ 20 μL·s^−1^. For the evacuation of analyte solution from the measuring system, a sterile silicon pipe from the peristaltic pump was inserted into the cell. The solution evacuation rate was adjusted in such a way that the cell was always filled with the analyzed solution. The solution evacuation rate was ~ 20 μL·s^−1^. The total time of protein fishing from the analyzed solution was ~ 1.5 h.

Protein fishing was followed by a washing procedure. For that, 15 mL of deionized water was pumped through the cell with a peristaltic pump. After that, the AFM chip with fished‐out protein was removed from the system. AFM visualization, counting of fished‐out objects in the sensor area of the surface and MS analysis for the identification of these objects were performed.

The AFM fishing experiments were carried out with the use of an external voltage source. In the experimental series a thin Pt electrode was immersed in the cell. The distance between the Pt electrode and the AFM chip surface was 2 mm. For stimulation of AFM fishing with external voltage, pulsed type AC voltages with a superimposed DC voltage were applied to the AFM chip. The superimposed DC voltage was 5 V. The external voltage was applied to the HOPG plate (which was one of the electrodes), while the Pt electrode was grounded. Pulses of AC voltage had ± 5 V amplitude, 1 ns rise time and 1 kHz frequency (a nanosecond pulsed AC electric field). The voltage was maintained using a Rezonans PG‐872 (Rezonans, Moscow, Russia) pulse generator power source.

Control experiments were carried out analogously to the technique of protein detection, but protein‐free water instead of protein solution was added to the measuring cell.

The design of the experiments was as follows. The experimental set‐up was assembled, and a pipe with a tip was installed into the system. Then, a series of experiments including one control and two working experiments with the use of protein solutions was carried out. A new AFM chip with immobilized avidin was used in each experiment. After that, the pipe and the tip were replaced with new ones, and the next series of experiments was carried out. In this way, six control experiments and three experiments with each protein solution with concentration from 10^−15^ to 10^−18^ m were carried out.

#### AFM visualization and counting of protein molecules on the surface

After protein fishing, the AFM images of the sensitive area of the AFM chip surface were collected using a Dimension 3100 (Veeco Instruments, Town of Oyster Bay, NY, USA) atomic force microscope and NTEGRA Aura AFM (NT‐MDT, Zelenograd, Russia). The AFM scanning was performed in air in tapping mode with the use of PPP‐NCH AFM‐probes (Nanosensors Inc., Neuchâtel, Switzerland); the curvature radius of these probes did not exceed 10 nm. Scan size was 5 × 5 μm, the number of scans was no less than 16 for each area, and the total scanned area *S*_scan_ = 400 μm^2^, that is *k* = *S*_scan_/*S* = 400/8000 (*k* is share of scanned area; *S* is total sensor area of the AFM chip), ~ 5% of the total sensor area of the AFM chip. Image analysis, counting of the registered objects and measurement of their heights was carried out using software developed at IBMC RAMS (Moscow, Russia) for treatment of AFM images (Rospatent registration no. 2010613458, 26 May 2010). Height (*h*) was the main criterion for measurements of molecular size, and the distribution of counted objects with heights ρ(*h*) was obtained 17, 18.

#### MS identification of proteins fished out onto the AFM chip surface

Trypsinolysis of proteins fished out using electrical AFM fishing was carried out directly on the AFM chip surface using a standard technique at constant temperature (42 °C) and ~ 100% air humidity. The incubation solution for trypsinolysis contained 100 mm bicarbonate buffer (pH 7.4), 10% acetonitrile, 1% glycerol and porcine trypsin in proportion to the target protein 1 : 50 14, 15. After the trypsinolysis, the trypsinolytic mixture containing peptide fragments was applied to a mass analyzer to perform the protein identification.

### SRM measurements

All liquid chromatography–SRM measurement experiments were performed on an Agilent 6490 (Agilent Technologies, Santa Clara, CA, USA) mass spectrometer coupled on‐line to an Infinity Series 1260/1290 chromatography system (Agilent). The instrument operated at positive ionization mode in a non‐scheduled SRM design. The drying gas (nitrogen) temperature was 280 °C, the drying gas flow was 14 L·min^−1^, the sheath gas (nitrogen) temperature was 250 °C, the sheath gas flow was 11 L·min^−1^, the capillary voltage was 4000 V, the nozzle voltage was at 900 V, the fragmentor voltage was 380 V and the cell accelerating voltage varied from 4.8 to 5.4 V depending on the target peptide; the full cycle time was 794.5 ms. The selected target peptides were LGEYGFQNALIVR with precursor ion *m*/*z* = 740.40; YLYEIAR with precursor ion *m*/*z* = 464.25; AEFVEVTK with precursor ion *m*/*z* = 461.75; and QTALVELLK with precursor ion *m*/*z* = 507.81. The MS1 and MS2 isolation windows were set at unit resolution (± 0.3 amu).

Peptides after digestion with trypsin were loaded in a volume of 4 μL onto the Eclipse Plus‐C18 RRHD column (Agilent) (2.1 × 100 mm, 1.8 μm particle size) at a flow rate of 100 μL·min^−1^ in 95% mobile phase A (0.08% formic acid, 0.01% trifluoracetic acid) and 5% of mobile phase B (0.08% formic acid, 0.01% trifluoracetic acid in 80% acetonitrile). The elution gradient was as follows: increasing from 5% to 10% of B for 2 min, linear elution from 10% to 60% of B (2–40 min), rapid increasing of B to 97% for 4 min (40–44 min), washing of the column at 97% of B for 5 min (44–49 min) and column reconstitution to initial conditions from 97% to 5% of B for 3 min (49–52 min). The post‐acquisition column equilibration was run for 7 min.

## Results

### Results of AFM chips surface visualization after avidin immobilization

**Figure 1:**
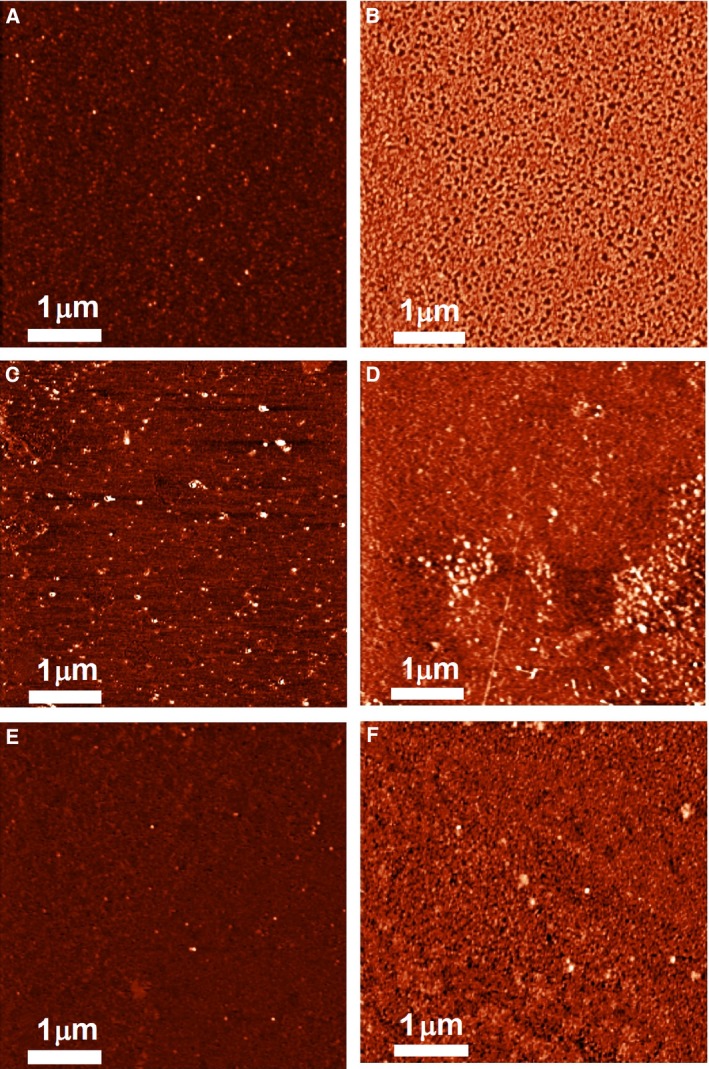
Examples of an AFM images of the chip surface. (A) After avidin immobilization. (B) After incubation in water (control experiment). (C–F) After incubation in biotinylated BSA solution of concentration 10^−15^ m (C), 10^−16^ m (D), 10^−17^ m (E) and 10^−18^ m (F). Scan size, 25 μm^2^; *Z*‐scale, 0–3.5 nm (A), 0–6 nm (B), 0–14 nm (C), 0–10 nm (D,E); 0–7 nm (F).

**Figure 2:**
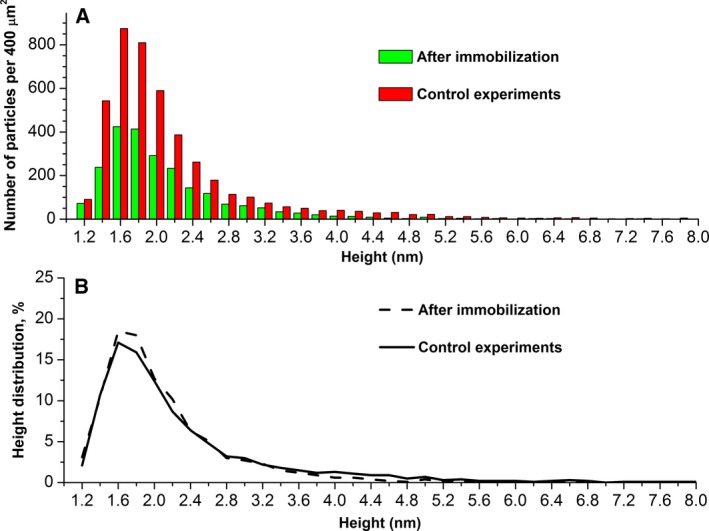
Results of processing of AFM data obtained after avidin immobilization (green bar, dashed line) and in control experiments (red bar, solid line) after the incubation of chips in water. (A) Height histogram of the visualized particles (the number of particles per scan area 400 μm^2^); (B) Density function ρ(*h*) of the visualized particles.

Atomic force microscopy chips with immobilized avidin molecules were prepared according to the technique described in ‘Materials and methods’. After the immobilization and formation of the sensor area with PTFE film, the surface of each AFM chip was visualized by AFM. An example of the image obtained is shown in Fig. 1A. As shown, after the immobilization a layer of particles was observed on the surface, along with separate compact objects with heights up to 4 nm. The number of particles in the layer with height ≥ 1 nm per 400 μm^2^ was, for individual samples, from ~ 500 to ~ 2500. For all AFM chips used in the experiment (18 samples), the distribution function of visualized objects ρ(*h*) was plotted (Fig. 2, dashed line) following the processing of the AFM scanning data for the surface with immobilized avidin. The obtained range of heights of the visualized objects corresponded to the data obtained previously for the fishing out of avidin molecules onto the chemically activated surface 8. Therefore, one can state that following the immobilization procedure, a layer of avidin biomolecules with separate particles with heights up to 4 nm is formed on the AFM chip surface, and such chips can be used for biospecific fishing and for control experiments.

### Results of control experiments

Control experiments were carried out to determine the characteristics of non‐specific objects adsorbed onto the surface of the AFM chip with immobilized avidin after the fishing procedure. The experiments were carried out according to the above‐described technique with the use of water without target molecules (BSA biomolecules). An example of the AFM images obtained is presented in Fig. 1B. As seen from this image, the surface topography had not changed significantly in comparison with the initial state after the immobilization procedure (compare with Fig. 1A). The processing of AFM scanning data showed that the normalized number of particles made up between ~ 1000 and ~ 3000 per 400 μm^2^ in individual experiments. After that, AFM data processing was performed in the aggregate for all control experiments (six samples), and the distributions of visualized particles with height ρ(*h*) were plotted (Fig. 2, solid line). As seen from Fig. 2A, the number of particles per scanned area was increased compared with the initial state of the chip after avidin immobilization (compare with Fig. 2A, dashed line). Thus, non‐specific sorption of the particles onto the surface was observed. However, the heights of the adsorbed particles were below 4 nm, which was graphically represented in the distribution function of visualized particles ρ(*h*) (Fig. 2B, solid line).

### Results of AFM chips surface visualization after protein fishing

**Figure 3:**
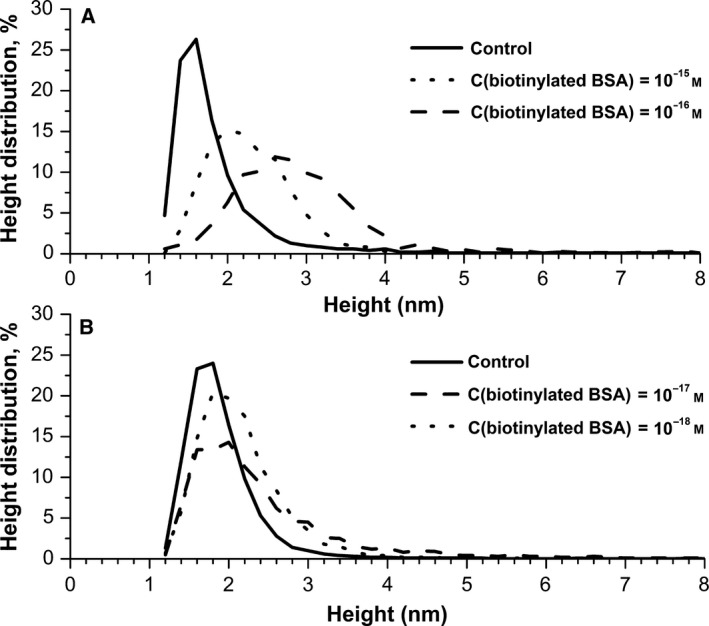
Results of processing of AFM data obtained after the incubation of chips in biotinylated BSA solution and in control experiments. Density function ρ(*h*) of the visualized particles. Protein concentration: (A) 10^−15^ and 10^−16^ m; (B) 10^−17^ and 10^−18^ m.

Experiments were carried out according to the AFM fishing technique described in ‘Materials and methods’ with the use of aqueous solutions of biotinylated BSA. Figure 1C–F shows examples of the AFM images obtained. As seen from these images, the surface topography had changed in all cases: objects with heights exceeding 2 nm were visualized on the surface. The processing of AFM scanning data showed that the normalized number of particles made up from ~ 1300 to ~ 6600 per 400 μm^2^ in individual experiments. Based on processing of AFM scanning data, the distribution function of visualized objects ρ(*h*) was plotted. Examples of the plotted distributions obtained in two experimental series are presented in Fig. 3A,B. Figure 3A shows an example of the control experiment and of working experiments from the same series with 10^−15^ and 10^−16^ m protein solutions. The normalized number of visualized objects in this series made up in the control experiment 2561 particles and in the working experiments 2226 and 1403 particles with 10^−15^ and 10^−16^ m protein solutions, respectively. Figure 3B shows an example of the control experiment and of working experiments from the same series with 10^−17^ and 10^−18^ m protein solutions. The normalized number of visualized objects in this series made up in the control experiment 2120 particles and the in working experiments 6614 and 5291 particles with 10^−17^ and 10^−18^ m solutions, respectively. As seen from Fig. 3, the objects visualized on the AFM chip surface after the fishing procedure (dashed lines) have in all cases broader distributions with height than the objects visualized on the surface in the control experiment (solid line).

### Results of MS identification

Mass spectrometry analysis was carried out for all samples obtained in the experimental series after the AFM fishing of biotinylated BSA from 10^−15^ to 10^−18^ m solutions and in control experiments. In MS measurements, the content of four unique tryptic peptides of BSA (LGEYGFQNALIVR, *m*/*z* = 740.40; YLYEIAR, *m*/*z* = 464.25; AEFVEVTK, *m*/*z* = 461.75; and QTALVELLK, *m*/*z* = 507.81) in the tested samples was analyzed by SRM.

**Figure 4:**
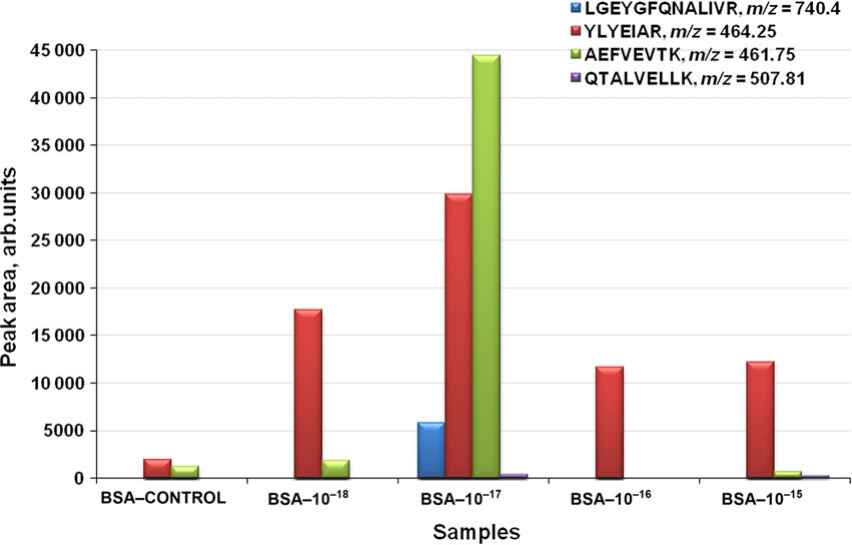
Chromatographic peaks of four unique peptides of biotinylated BSA obtained for an experimental series including AFM‐SRM analysis of the control sample (BSA–Control) and four working AFM samples – BSA–10^−18^, BSA–10^−17^, BSA–10^−16^ and BSA–10^−15^ – after the incubation in biotinylated BSA solutions with 10^−18^, 10^−17^, 10^−16^ and 10^−15^ m concentration, respectively. The values are presented in a.u. of chromatographic peak area for the biotinylated BSA peptides indicated with color bars (see key).

Working samples representing washings from the surface of AFM chips after their incubation in target protein solution in the 10^−15^–10^−18^ m concentration range were analyzed. Results of one series of AFM–MS measurements are presented in Fig. 4. The control sample represented the AFM chip with immobilized avidin molecules. MS analysis of the control sample was carried out after the incubation of the chip in solution without biotinylated BSA. However, in the control sample (BSA–Control) noise MS signals corresponding to two peptides of biotinylated BSA, YLYEIAR and AEFVEVTK, with the peak area values 1943 and 1232, respectively, were registered. To differentiate the noise signals from the target ones, the areas of the registered peaks in the range 0–3000 arbitrary units (a.u.) were attributed to the noise signal, which is nearly twofold greater than the signals registered in the control sample.

As seen from Fig. 4, MS signals obtained for working samples are at least an order of magnitude higher than the noise signals obtained for the control sample. In all the four samples, the biotinylated BSA peptide YLYEIAR (*m*/*z*: 464.25) was detected. It is noteworthy that for the working samples, incubated in 10^−15^, 10^−16^ and 10^−18^ m biotinylated BSA solutions, the chromatographic peak areas were comparable. Of interest is the working sample incubated in 10^−17^ m biotinylated BSA solution, in which three peptides of biotinylated BSA with peak areas differing widely from those for other working samples were revealed (see Fig. 4).

## Discussion

The results of AFM analysis have indicated that after the AFM‐based fishing with electrical stimulation procedure, the objects whose height increase contributed to the right wing of the ρ(*h*) distribution are visualized on the surface of AFM chips with immobilized avidin (Fig. 3). These objects can be attributed to avidin–biotinylated BSA complexes, since such a contribution is not observed in control experiments. Non‐specific sorption of particles does not influence the results of the experiments. As shown in Fig. 2, the shape of ρ(*h*) dependences coincides with the data from control experiments and with the data on AFM visualization of the surface after avidin immobilization. That is, the height of non‐specifically adsorbed particles is less than that of complexes formed as a result of biospecific interaction BSA‐biotin–avidin.

According to the AFM data, the number of AFM visualized objects after the fishing procedure under the conditions described depends on the concentration of the protein solution. As shown in the ‘Results’ section on two experimental series, the largest number of fished‐out particles was registered for fishing from 10^−17^ m protein solution, and the least amount for fishing from 10^−16^ m solution. According to the results of other series, the normalized number of objects varied, but the tendency *n* (10^−17^ m) > *n* (10^−18^ m) > *n* (10^−15^ m) > *n* (10^−16^ m) persisted. The same tendency was observed by us during the non‐specific protein fishing for the test solution injection into the measuring cell 5.

The results of the SRM analysis fully confirm quantitative estimates of the number of visualized particles in the AFM analysis. So, in accordance with the intensities of the obtained MS signals and according to the number of identified unique BSA‐biotinylated peptides in the working samples one can form an evaluation order for the content of target BSA‐biotinylated molecules on the AFM chip surface: *n* (10^−17^ m) > *n* (10^−18^ m) > *n* (10^−15^ m) > *n* (10^−16^ m).

Only in the working sample BSA–10^−17^ (as defined in Fig. 4 legend), three peptides of the target protein were identified after the incubation in 10^−17^ m biotinylated BSA solution; in the other working samples, it was possible to identify only one peptide, YLYEIAR.

The values of the registered peak areas for the YLYEIAR peptide are distributed as follows: BSA–10^−17^, 29 826 a.u.; BSA–10^−18^, 17 712 a.u.; BSA–10^−15^, 12 169 a.u.; BSA–10^−16^, 11 657 a.u. The greatest differences in the values of the recorded signal areas were observed between the following samples: BSA–10^−17^ and BSA–10^−18^, 45% reduction; BSA–10^−18^ and BSA–10^−15^, 31% reduction. The registered area for the working sample BSA–10^−16^ was reduced insignificantly, by 4%, compared with the BSA–10^−15^ sample. The results of MS measurements correspond to the AFM data obtained. Therefore, one can state that biotinylated BSA can be detected by the combined AFM–SRM method in solutions at concentrations from 10^−15^ to 10^−18^ m upon application of the flow system for sample solution feed.

Previously we have shown that, according to theoretical estimates, by taking into account only the diffusion model, it is impossible to detect protein in experimental conditions at 10^−15^–10^−18^ m concentrations 5. A significant discrepancy in the theoretical and experimental data as to the time required for fishing the proteins out of low concentration solutions was discussed earlier 5. The fact is that the liquid flow leads to an electric potential at the phase boundary 16. In this situation, the magnitude of the electric potential depends on the flow rate. The measurement system for AFM‐based fishing, as used in this study, contains several such interfaces: protein solution–walls of solution feed system; protein solution–PTFE cell walls; protein solution–HOPG surface; protein solution–PTFE film surface. The joint effect of the electric fields arising on these interfaces due to solution flow leads to significant reduction of time required for protein detection in low concentration solution. The triboelectric effect is thus one of the factors determining efficiency of protein fishing in experimental conditions used previously 5 and in the present study. The charges generated upon solution input can be captured by the protein molecules, which turn into more charged particles. It appears therefore that the lower is the protein solution concentration, the greater is the charge induced on one protein molecule. Consequently, one can assume that the fishing efficiency is directly connected with the value of the charge induced on a protein molecule. Hence, an inverse dependence should be observed: the fishing efficiency must increase with a decrease in the protein concentration. The data obtained in the experiments in the present study and in 5 confirm the above‐made assumption.

Another factor influencing the protein fishing efficiency (as is indicated by the results obtained in this study and in 12) is an external electric field. To provide the detection efficiency, pulsed electrical stimulation of AFM‐based fishing is required. Electrical stimulation of protein fishing in the flow mode was implemented with nanosecond pulses, i.e. by applying a pulsed voltage of a rectangular shape with a rise time of 1 ns. Such pulsed electrical stimulation of AFM‐based fishing is possibly conditioned by a local increase of electric field gradient and induced magnetic field on HOPG in the vicinity of the mica surface. The effect of increasing electric field gradient can lead to possible excitations of plasmons in graphite, constituting the layers of highly conductive graphene flakes 19, and eddy currents can also be generated on the graphite surface 20. Besides, it should be noted that dielectric properties of the mica surface in the mica–graphite structure in their turn can influence the possibility of plasmon generation, and also change due to the changes in water structure on the surface of mica with immobilized probe molecules, as well as other electrokinetic effects. Intensive local gradients of electric fields can cause polarization of protein molecules by intensive induced magnetic field influences on the spin state of protein–water molecule complexes, and, hence, stimulate their motion towards the mica–graphite structure from the analyte solution. It has to be taken into account that the influence of the electromagnetic field in water at the high rate of the pulse rise is obviously not limited by the Debye length, which is ~ 2 nm in 10 mm potassium phosphate buffer 20 and able to reach hundreds micrometers in aqueous medium 21.

It has to be noted that in this study the stimulation of AFM‐based fishing with AC electric field was observed at its front rise time and that it was in the order of 1 ns. At the same time, the field strength was 10–100 times lower than the values commonly used in microfluidic devices.

The advantage of the proposed approach is the application of low strength AC field and, accordingly, the mandatory use of capillaries to create strong electric fields. That is, fishing with electrical stimulation may be implemented in the systems with large channels, and this is the preferred technological solution for proteome and diagnostic research in analysis of biological fluids.

## Conclusion

It was shown that biospecific detection of protein can be carried out in solution at ultra‐low concentrations from 10^−15^ to 10^−18^ m. Electrical stimulation – pulsed electric field applied to the AFM chip placed onto HOPG – is required to fish out the target protein. Efficient fishing of protein is demonstrated on the application of a conventional system of sample feed with a peristaltic pump. The results obtained in this study can be used in routine bioanalytical practice and in the development of highly sensitive analytical systems.

## Author contributions

TOP and YDI designed and conceived the research and wrote the paper. TOP, KAM, ALK, ATK, SLK and RAG performed research and acquired the data. TOP, ALK, VYT, VSZ and SLK analyzed and interpreted the data.
